# Dynamic Manugraphy as a Promising Tool to Assess the Outcome of Limited Aponeurectomy in Patients With Dupuytren's Contracture

**DOI:** 10.3389/fmed.2020.604891

**Published:** 2021-01-12

**Authors:** Angelina Garkisch, Thomas Mittlmeier, Axel Kalpen, Marion Mühldorfer-Fodor, Dagmar-C. Fischer, Alice Wichelhaus

**Affiliations:** ^1^Department of Traumatology, Hand- and Reconstructive Surgery, Rostock University Medical Centre, Rostock, Germany; ^2^Novel Biomechanics Laboratory, Munich, Germany; ^3^Clinic for Hand Surgery, Rhön Klinikum AG, Bad Neustadt, Germany; ^4^Department of Pediatrics, Rostock University Medical Centre, Rostock, Germany

**Keywords:** dynamic manugraphy, physiological gripping, grip force, limited aponeurectomy, hand function

## Abstract

**Background:** Dupuytren's contractures interfere with physiological gripping. While limited aponeurectomy is an accepted treatment modality to restore finger mobility, methods to objectify functional outcome beyond determination of the range of motion are scarce.

**Methods:** Patients with Dupuytren's contracture being scheduled for unilateral limited aponeurectomy were invited to participate. Clinical data were gathered prospectively by chart review and interview. The DASH-score and flexion contracture for fingers were registered prior to surgery, 3 and 6 months afterwards. At the same time, dynamic manugraphy for simultaneous recording of the grip pattern and forces generated by the affected hand and anatomic areas (i.e., thumb, index finger, middle finger, ring finger, little finger and palm) were performed. All findings obtained during the follow-up period were compared to the situation at baseline. Comparison between paired samples was done using Wilcoxon rank test. All *p*-values are two-sided and *p* < 0.05 was considered to be significant.

**Results:** Out of 23 consecutively enrolled patients, 19 (15 men, 4 women) completed follow-up examinations. Manugraphy confirmed the impairment of physiological gripping with concomitant pathological load distribution at base line. Limited aponeurectomy significantly reduced flexion contractures. However, the DASH-score remained at an excellent level in one patient, indicated improvement in 11 and worsening in seven patients. Six patients had lower grip force at t_6_ compared to the preoperative condition, although the preoperative flexion contracture (≥110°) was considerably improved in all of them. In four of those, the DASH-score improved while it turned worse in two of them. The force of surgically treated fingers remained unchanged in three patients while it was improved and worsened in half of the remaining patients, respectively. Manugraphy revealed physiological gripping by enlargement of contact area and higher force transmission by the fingertips in 10 of 12 patients with constant or even improved DASH-score and in three of seven patients with a worsened DASH-score.

**Conclusions:** Assessing the reduction of flexion contracture and grip force alone is not sufficient to comprehensively reflect the functional outcome of aponeurectomy for Dupuytren's disease. Visualizing physiological grip pattern provides an additional tool to objectify the success of surgical treatment.

## Introduction

Dupuytren's contracture is a benign, albeit irreversible progressive disease of the palmar aponeurosis that causes increasing flexion contractures, progressive flexion deformities and a decreased range of motion of the fingers (1, 2). It is a common disorder of connective tissue, affecting 5–25% of individuals of European descent and genome-wide association study revealed several relevant loci (3–7). Furthermore, patients with diabetes mellitus, liver diseases or epilepsy as well as those with chronic exposure to strong hand-arm vibrations, high levels of alcohol consumption and smoking are at risk to develop such flexion deformities (1, 2, 8–12). Dupuytren's contracture preferentially affects the fourth and fifth fingers and almost always interferes with both, the ability to grip via controlled formation of a fist and with grip strength. Since gripping is the main function of the hand, any functional impairment will impact on activities of daily life rather than being mainly a cosmetic problem (13). However, individual perception of the severity of the disease and the concomitant request for therapy is rather variable. Although timing of surgery relative to the time course of the disease is still a matter of discussion, outcome was shown to be best when the contracture of the proximal interphalangeal joint (PIP) is between 15° and 30° (14–16). Restoration of functional extension is most frequently achieved via limited aponeurectomy, i.e., macroscopically affected tissue of the palmar aponeurosis as the culprit of flexion deformities is removed (17–19).

Although gripping is such an important ability for almost all aspects of life, methods for the assessment of this function are scarce. Instead, patient reported outcome measures and the range of motion are thus far used to quantify the severity of the disease and the impact of treatment (20).

Recently, the manugraphy system has been introduced as a sensitive technique for time and space resolved analysis of gripping (21, 22). A mat with an inbuilt array of sensors designed to translate pressure into an electronic signal is wrapped around a cylinder. This design allows simultaneous recording of the contact area of the inner surface of the finger involved in gripping together with the forces generated by all areas of the hand and fingers during physiological gripping (23). We hypothesized, that manugraphy will not only be especially suited for the objective assessment of the functional impairments related to Dupuytren's contracture prior and after surgery, but will enable us to evaluate whether or not the grip force has improved 6 months after limited aponeurectomy. Furthermore, we hypothesize that the outcome after limited aponeurectomy will be reflected by the DASH-score and this changes are expected to correlate with grip pattern.

## Methods

### Patients

Patients presenting between 05/2016 and 12/2018 with primary or recurrent Dupuytren's contracture and being scheduled for unilateral limited aponeurectomy were eligible and invited to undergo dynamic manugraphy prior to surgery and at pre-defined intervals afterwards. Patients with cognitive impairments, severe rheumatic disease and neurological disorders leading to flexion deformities of the fingers were not eligible. The study received appropriate ethics committee approval from the institutional Ethics Committee in accordance with the Declaration of Helsinki (approval number: A 2016 0009) and all patients gave written and informed consent for participation. Limited aponeurectomy was performed according to standard procedures during regional anesthesia and concomitant ischemia of the hand in our clinic. In particular, a zig-zag volar-digital incision over the contracture was followed by removal of macroscopically affected tissue (18, 24). When full extension of the respective fingers was possible, ischaemia was resolved prior to performing a z-plasty and suturing with simple interrupted stitches. A compression bandage was applied and controlled 24 h later. Elevation, cooling and an early initiation of unlimited functional rehabilitation by means of ergo- or physio-therapy was recommended.

### Study Investigation

Patients were examined prior to surgery (t_0_) and follow-up examinations were scheduled 3 (t_3_) and 6 (t_6_) months later with an allowed deviation of ±14 days. Demographic and clinical data including history of disease and the number of affected fingers were gathered by interview. The DASH-score (Disabilities of the Arm, Shoulder and Hand) was used to assess functionality of the upper limb from the patient's perspective (25). The DASH-score is combined of 30 questions, each using a Lickert scale to indicate the severity of symptoms from best (1 point) to worse (5 points) and the final results are expressed on a scale from 0 (best outcome) to 100 (worst case) (26, 27). Study examinations of both hands consisted of a standardized assessment of the flexion contracture by the same examiner (AG). A precision goniometer with an analog display (Sammons Preston, Bolingbrook, IL) was used to quantify the angle of contraction for each joint of the affected fingers and per finger the results were summed up.

**Figure 1:**
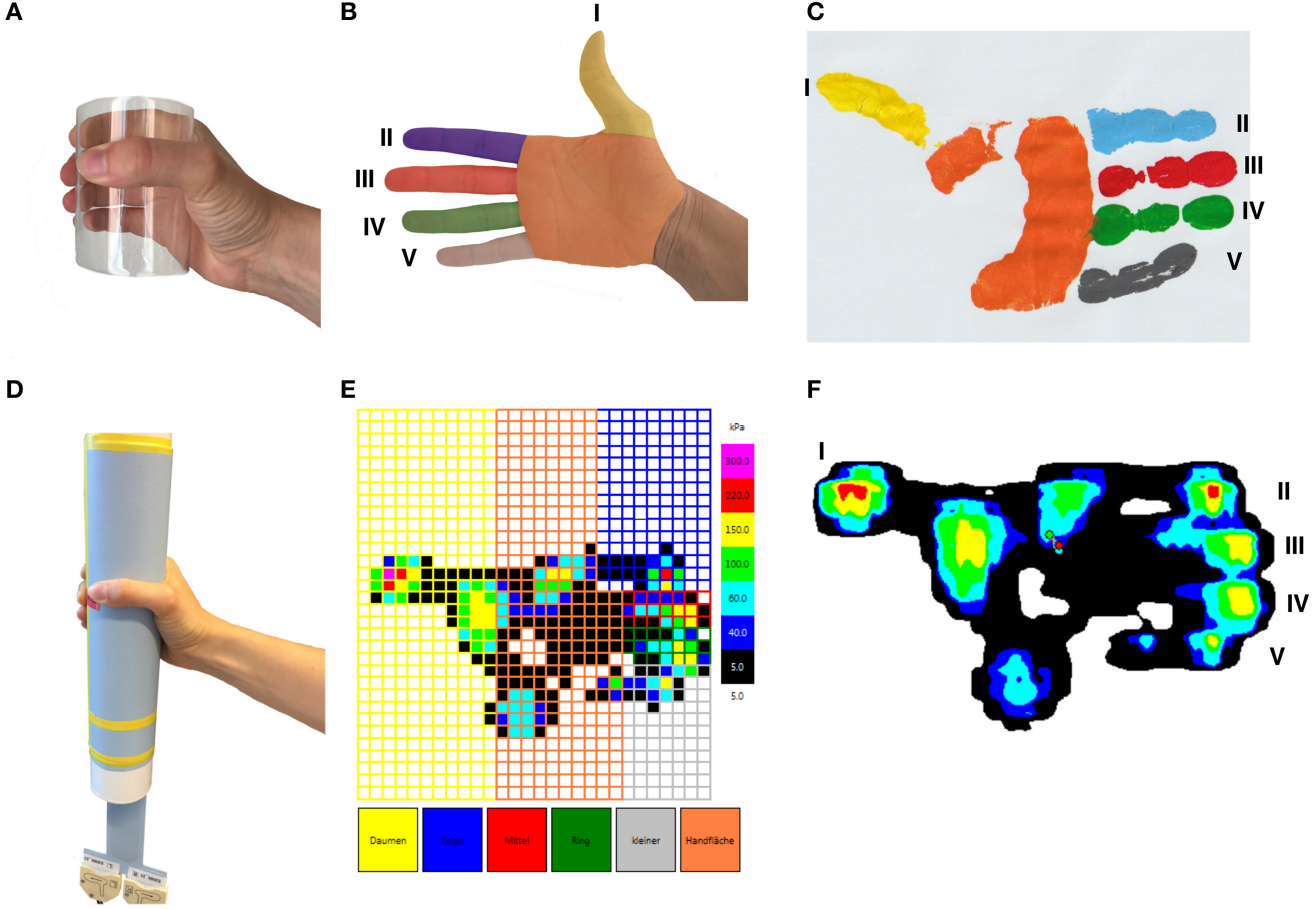
Schematic visualization of a right hand- and fingerprint. A hand gripping a cylindrical object **(A)**, six anatomical areas of the hand (thumb, index finger, middle finger, ring finger, little finger, palm) **(B)**. Visualization of a hand print after gripping a cylindrical object **(C)**. Positioning of the hand during the cylinder grip **(D)**. An example of the results of a physiological hand print gained by the manugraphy system depicted as pressure **(E)** and isobars **(F)**. Sensors covered by thumb (I), index finger (II), middle finger (III), ring finger (IV), little finger (V), and palm are indicated.

Manugraphy (manugraphy® system, novel, Munich, Germany) was utilized to assess grip strength and to visualize the load distribution of the hand during gripping (21, 28) essentially as described. A cylinder (circumference: 200 mm, diameter: 64 mm) surrounded by a mat with in-built pressure sensors (2 per cm^2^) and an interface for data transfer (sampling rate 20 Hz) to the proprietary software for data acquisition and analysis are at the heart of the system. Gripping of the cylinder is equivalent to the application of pressure to the sensors within this particular area and the generation of a time and space resolved electronic handprint. Beside quantitative information on the total force during gripping together with the forces applied by the thumb, each of the fingers and the palm of the hand, the load distribution of the hand is visualized by means of a colored scale (Figure 1) (29). During the measurements, the patient was positioned on a chair with the upper arm adducted, elbow 90° flexed and the hand loosely gripping around the cylinder hanging with the long axis in a vertical orientation (30). Investigations started always with the non-operated hand and after familiarization with the system, three sequences of gripping with maximum force (5 s) followed by loosely gripping (10 s) were recorded. To assure reproducible conditions, an audio file gave instructions to grip with maximum force and to release grip force subsequently. The mean of these measurements was used for statistical analysis. For every patient, the data obtained at baseline served as reference.

### Statistical Analyses

Apart from the proprietary software of the manugraphy system, SPSS statistical package 25 (SPSS Inc. Chicago, Illinois, USA), and Sigma Plot Version 10 (Jandel Scientific Inc.) were used. Due to the rather low sample number all data are given as median and range and the Wilcoxon rank test was employed for comparison between paired samples. All *p*-values were two-sided and a *p*-value below 0.05 was considered significant.

## Results

### Patients Characteristics

Out of 28 patients eligible for participation, 23 patients gave written informed consent and underwent baseline investigations prior to surgery. Data from follow-up examinations were obtained from 19 patients (15 men, 4 women) and these were considered for analysis. Drop-outs were due to non-attendance or loss of contact. Demographic and clinical data of the patients are given in Table 1. None of them complained about a contracture of the thumb. Surgery of the index finger was performed in 2 (6.9%), of the middle finger in 4 (13.8%), of the ring finger in 9 (31%) and of the little finger in 14 patients (48.3%). Limited aponeurectomy was solely applied to the middle finger in two patients, to the ring finger in two patients and to the little finger in eight patients. Surgery of the ring and little fingers was done in five patients, while limited aponeurectomy was performed of three (index, middle, and ring finger) and four fingers in one patient each.

**Table 1:** Characteristics of the patients.

	**Enrolled (19m/4f)**	**Complete examination (15m/4f)**
**Anthropometric data**		
Age (years)	61 (38–79)	62 (38–79)
Height (cm)	180 (156–187)	180 (156–187)
Weight (kg)	84,5 (52–120)	82 (52–120)
BMI (kg/m^2^)	26 (20–37)	26 (20–37)
Dominant hand (both/left/right)	2/3/18	2/1/16
Operated hand (left/right)	14/9	13/6
Contracture (unilateral/bilateral)	8/15	6/13
Surgery for recurrence (*n*)	5	4
**Clinical data**		
Fractures of the upper limb [*n*]^*^	10/6	10/6
Other injuries/pathologies of the upper limb [*n*]^#^	4/2	4/2
Type 2 diabetes mellitus [*n*]	4	3
Hypertension [*n*]	4	3
Bronchial asthma [*n*]	2	2
Angiopathy [*n*]	2	2
Alcohol abuse [*n*]	2	2
Depression [*n*]	1	1
Restless leg syndrome [*n*]	1	1

* *Fractures of the upper limb: fractures of the phalanges, distal radius fracture, olecranon fracture, proximal humerus fracture*.

# *Other injuries/pathologies of the upper limb: cutting damages, de Quervain's tendinitis, arthrosis of the shoulder*.

### Study Examinations

#### DASH-score and Resolving of the Flexion Contracture

The median DASH-score dropped from median 11 (range 0–60) at time of enrolment to 5 (range 0–54) 6 months after surgery. The DASH-score remained constant and indicated virtually no disabilities of the arm, shoulder or hand in one patient. The score revealed an improvement of the condition in 11 patients and worsening in seven patients, respectively (Table 2; Supplementary Table 1). Patients with an improved DASH-score had a significantly improved flexion contracture at t_6_ in the ring finger (*p* = 0.042) and little finger (*p* = 0.008).

**Table 2:** DASH-score and flexion contracture of the diseased fingers prior to surgery and 6 months afterwards.

	**DASH**		**Affected fingers**	**Flexion contracture**	
	**t_**0**_**	**t_**6**_**		**t_**0**_**	**t_**6**_**
Overall (*n* = 19)	11 (0–60)	5 (0–54)	IF (*n* = 2) MF (*n* = 4) RF (*n* = 9) LF (*n* = 14)	60/85^#^ 35 (30–120)^*^ 65 (10–120)^*^ 98 (40–145)^*^	10/50^#^ 8 (0–80)^*^ 0 (0–95)^*^ 30 (0–80)^*^
Improvement (*n* = 11)	23 (2–60)	5 (0–34)	IF (*n* = 1) MF (*n* = 2) RF (*n* = 5) LF (*n* = 9)	85^#^ 30/40^#^ 30 (25–120)^*^ 110 (40–145)^*^	10^#^ 0/15^#^ 0 (0–5)^*^ 25 (0–45)^*^
Worsening (*n* = 7)	6 (0–13)	19 (4–54)	IF (*n* = 1) MF (*n* = 2) RF (*n* = 5) LF (*n* = 4)	60^#^ 40/120^#^ 95 (55–120) 98 (60–115)	55^#^ 0/80^#^ 65 (65–95) 38 (20–80)
Unchanged (*n* = 1)	1	1	RF (*n* = 1) LF (*n* = 1)	10^#^ 75^#^	0^#^ 30^#^

* *indicates significant differences between data obtained at t_0_ and t_6_ (p < 0.05)*.

# *In case two or less patients were operated on a particular finger, individual data instead of median and range is given*.

The flexion contractures were reduced in six out of seven patients claiming on a worsened DASH-score while one patient experienced an early recurrence with a flexion contracture greater than before. Two patients had a severe preoperative flexion contracture of 120° in 1 finger and further fingers were affected. In four patients with one finger affected in each, the contractures were improved and a residual contracture of 45°, 0°, 30°, and 20° remained.

#### Grip Force and Load Distribution of the Hand Relative to the DASH-score

The force per anatomical area of the hand tended to be lower at t_3_, even for the thumb, which was not surgically addressed. At t_6_, the average total grip force as well as the average force of the thumb and index finger reached almost preoperative values, while the force generated by palm, middle, ring, and little finger exceed preoperative values (Table 3). The difference was significant for the middle finger. However, looking at each finger treated, three had the same finger force at t_6_ than preoperatively, half of the rest had the finger force increased, half decreased. Six patients had lower total grip force at t_6_ compared to the preoperative condition. In all of them the preoperative flexion contracture, which ranged from 110° up-to 125°, was considerably improved. Four of them had the DASH-score improved, in two of them the DASH-score was worse. Neither in patients with an improved nor in patients with a worsened DASH-score a significant difference between grip forces at t_0_ and t_6_ was seen.

**Table 3:** Absolute grip forces generated by total hand as well as thumb, fingers and palm at t_0_, t_3_, and t_6_.

	**Absolute grip force [N] of the Hand, Thumb, Fingers, and Palm**		
	**t_**0**_**	**t_**3**_**	**t_**6**_**
Hand	362^*^(83−700)	321^*^(132−544)	353(202−718)
Thumb	114(28−210)	90(36−171)	104(67−229)
Indexfinger	54^*^(25−124)	49^*^(23−89)	53(24−107)
Middlefinger	49^*^(11−82)	40(19−86)	56^*^(27−109)
Ringfinger	26(0−85)	29(11−49)	36(16−84)
Littlefinger	18(0−43)	19(0−35)	20(0−37)
Palm	100(12−231)	70(39−170)	114(40−199)

* *indicates significant differences between data obtained at baseline and during follow-up examinations (p < 0.05)*.

**Figure 2:**
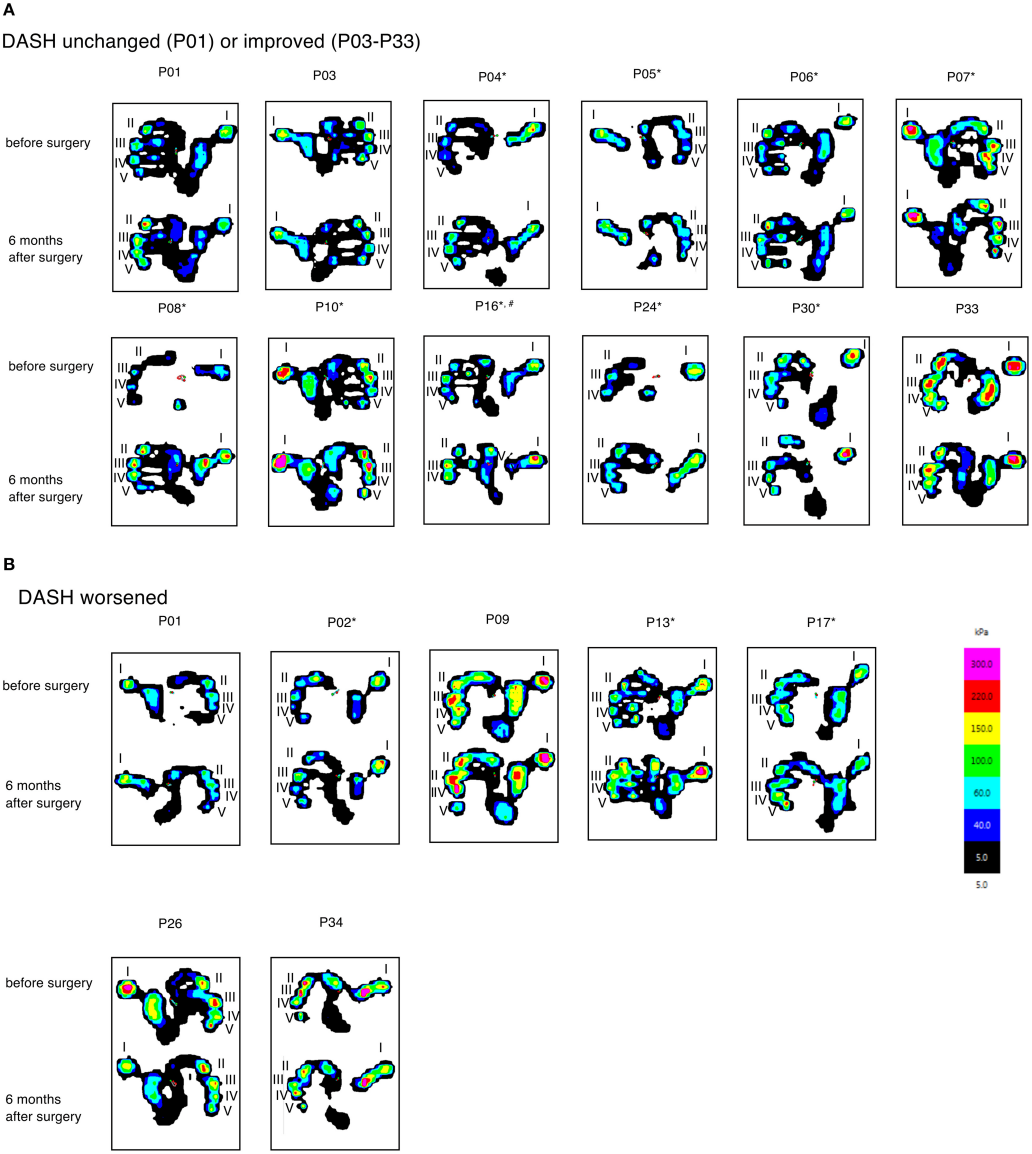
Hand- and fingerprints visualized as isobars obtained at t_0_ and t_6_ from patients with unchanged or improved **(A)** and worsened **(B)** DASH-score. The area covered by the respective part of the hand and the amount of pressure transmitted to the sensor in these particular regions are indicated by color. Asterix are used to indicate patients with improved gripping abilities, i.e., and increased force of the fingertips and an enlarged area covered during gripping.

For each patient, the load distribution of the hand prior to surgery and 6 months afterwards is presented in Figure 2. In general, planar application of the proximal and middle phalanx was impossible at time of enrolment. Resolving the flexion contracture results in almost all patients in an improved gripping at t_6_ by an enlarged contact area, especially at the base of the fingers and the hypothenar, and an increased transmission of force through the fingertips.

Finally, we investigated the changes of both, the grip pattern and the DASH-score taken in parallel prior to surgery and 6 months afterwards. Manugraphy revealed a more physiological gripping in nine of 11 patients with an improved and in three out of seven patients with a worsened DASH-score. In one patient with an already high DASH-score (0.83) the grip pattern at t_6_ was improved compared to the situation before surgery.

## Discussion

Dupuytren's contracture of one or more fingers are likely to interfere with gripping and may eventually lead to severe disabilities of the hand. Limited aponeurectomy is an established procedure for resolving of the contracture and restorage of the abilities for physiological gripping and grip strength. Thus far, outcome is rarely investigated in terms of functionality but the degree of the flexion contracture and patient reported outcome (PROM) measures like the DASH-score are used instead. While surgery was successful in resolving the flexion contracture in almost all patients, the DASH-score worsened in a reasonable number of patients. First of all, the DASH is a rather broad instrument and allows hardly to sort out whether the problems are due to an impairment of the arm, the shoulder or the hand (20). In fact, a previous fracture of the distal radius, the humerus or even other injuries of the arm or affected hand was reported from patients of either group. Although the DASH questionnaire is commonly used in patients with Dupuytren's disease, functional assessment is required to validate treatment efficacy even on an objective scale.

The present study is the first one employing manugraphy for the assessment of gripping in patients with Dupuytren's contracture prior and after unilateral surgery. Recently, we described significantly different grip forces with respect to sex and handedness (31). However, the limited number of patients enrolled in the current study did not allow to control for these confounders. Thus, we decided to investigate the outcome relative to the findings at baseline, i.e., every patient serves as his own control. This approach confirmed that it will take several months for the hand function to recover from surgery, i.e., the entire grip strength of the hand as well as the forces generated by thumb and index finger reached almost preoperative values 6 months after surgery. However, the grip strength of the middle finger, the ring finger, the little finger, and the palm was improved 6 months after removal of the contracture.

Patients with an improvement of the DASH-score had a significantly improved flexion contracture 6 months after surgery in the ring and little finger. This may suggest a better functionality of the hand due to improvement of the flexion contracture of the two ulnar fingers. However, neither in patients with an improved nor in patients with a worsened DASH-score grip strength was significantly changed after limited aponeurectomy. This notion is well in line with the recently reported loss of grip strength as assessed by the Jamar dynamometer and pinch strength relative to the contralateral hand in patients with recurrent contractures (32). Thus, flexion contracture and impaired gripping rather than grip strength might be the main problem of patients with Dupuytren's contracture.

While information on grip strength, even of individual fingers and the thumb can be obtained with less effort by utilization of an appropriate dynamometer, the entire strength of the manugraphy is to provide insight into the physiology of gripping. In fact, prior to surgery the vast majority of patients was unable to grip the cylinder efficiently due to the limited ability to extend the ulnar fingers (33). Accordingly, the contact area between the palm and the sensor mat is inadequately low and the process of gripping is rather unphysiological. Removal of the contracture restores the ability for physiological gripping and the majority of the patients covered a larger area of the sensor mat at t_6_. This clearly points to a discrepancy between the objective and subjective perception of the outcome. While seven patients claimed on worsening of the DASH-scores despite the fact, that the flexion contracture was reasonably reduced in six of them and manugraphy revealed a concomitant improvement of the gripping abilities in three of them. On the other hand, load distribution was unchanged and the force generated by the tips of the fingers was reduced in one patient each of those enjoying an improvement of the DASH-score. Beyond this, our findings are in line with the *quadriga phenomenon*, i.e., improving the abilities of one finger has the potential to improve the abilities of the adjacent fingers as well (34, 35). This effect is most likely due to the interconnectedness of the tendons of the flexor digitorum profundus (FDP) muscle and can decrease overall hand function. Consequently a restored range of motion of one finger due to limited aponeurectomy can result in a better functionality of adjacent fingers. In summary, subjective and objective outcome were concordant in 15 out of 19 patients. Therefore, we suggest manugraphy as a powerful tool to demonstrate the effects of the treatment to the patient. While this will certainly not turn an unfavorable outcome into a favorable one, it may help to objectify functionality of the hand even in the perception of the patient. Furthermore, manugraphy may aid to discriminate whether the discontent detected by means of the DASH-score might be rather unspecific or caused by an impairment of grip function.

While the strength of the present study is the longitudinal design with a comprehensive set of examinations prior to surgery and during a 6 months follow-up period, the rather low number of patients prevents discrimination between primary and recurrent disease or patients with uni- and bi-lateral contractures. In this regard, a carefully designed multicenter study is warranted to figure out the efficacy of treatment relative to the underlying conditions.

## Conclusion

In our knowledge, this is the first longitudinal study addressing the recovery of grip force and hand function by manugraphy in patients undergoing surgery for Dupuytren's contracture. To our surprise, it takes quite a long time for recovery of hand function, although grip force of the middle finger, ring finger, little finger, and the palm was improved already 6 months after limited aponeurectomy. Manugraphy revealed that the contact area between hand and cylinder to grip is increased 6 months after limited aponeurectomy. Furthermore, manugraphy can help to distinguish a general discontent of the patient from an actual impairment of grip function and to objectify the success of surgery.

## Data Availability Statement

The raw data supporting the conclusions of this article will be made available by the authors, without undue reservation.

## Ethics Statement

The studies involving human participants were reviewed and approved by Ethics Committee of Rostock University Medical Centre, St. Georg-Str. 108, 18055 Rostock (reference no. A 2016-0009). The patients/participants provided their written informed consent to participate in this study.

## Author Contributions

AW and TM designed the study. AW recruited the patients and performed limited aponeurectomy. AG acquired, analyzed, and interpreted the patient data and was a major contributor in writing the manuscript. D-CF supported data acquisition and drafting of the manuscript. MM-F and AK were involved in drafting the manuscript. All authors read and approved the final manuscript.

## Conflict of Interest

AK was employed by the company novel gmbh. The remaining authors declare that the research was conducted in the absence of any commercial or financial relationships that could be construed as a potential conflict of interest.

## Supplementary Material

Supplementary Table 1 Sex, flexion contractures of the operated fingers and grip strength of the anatomical areas as well as total grip strength represented for each patient before surgery as well as 3 and 6 months after limited aponeurectomy. Findings are grouped according to an unchanged, improved and worsened DASH-score, respectively.

## Abbreviations

DASH: Disabilities of the Arm, Shoulder and Hand
IF: index finger
LF: little finger
MF: middle finger
RF: ring finger.
